# An *in situ* forming gelatin-based hydrogel loaded with soluble amniotic membrane promotes full-thickness wound regeneration in rats

**DOI:** 10.22038/IJBMS.2024.74290.16140

**Published:** 2024

**Authors:** Mohammad Azimi-Alamouty, Mohammad amin Habibi, Amin Ebrahimi Sadrabadi, Zahra Jamalpoor

**Affiliations:** 1 Trauma Research Center, Aja University of Medical Sciences, Tehran, Iran; 2 Department of Tissue Engineering, Faculty of Basic Sciences and Advanced Technologies in Medicine, Royan Institute, ACECR, Tehran, Iran; 3 Iranian Tissue Bank and Research Center, Gene, Cell and Tissue Institute, Tehran university of Medical Sciences, Tehran, Iran

**Keywords:** Drug delivery, Growth factor, Hydrogel dressing, Skin tissue engineering, Wound healing

## Abstract

**Objective(s):**

Early effective treatment and appropriate coverage are vital for full-thickness wounds. Amnion membrane-derived products have recently emerged in tissue engineering. However, the optimal concentration, carrier for controlled release, and handling have remained challenges. This study aims to develop and optimize an *in situ* forming, amniotic-based hydrogel for wound healing.

**Materials and Methods:**

Here, a composite matrix was fabricated with gelatin hydrogel modified with methacrylate functional group conjugated (GelMA) and keratose (wt.1%), loaded with mesenchymal stem cells (MSCs, 1×10^5^ cell/ml) and optimized soluble amniotic membrane (SAM, 0.5 mg/ml). The physicochemical properties of the final subject were evaluated *in vitro* and *in vivo *environments.

**Results:**

The results of the *in vitro* assay demonstrated that conjugation of the methacryloyl group with gelatin resulted in the formation of GelMA hydrogel (26.7±1.2 kPa) with higher mechanical stability. Modification of GelMA with a glycosaminoglycan sulfate (Keratose) increased controlled delivery of SAM (47.3% vs. 84.3%). Metabolic activity (93%) and proliferation (21.2 ± 1.5 µg/ml) of MSCs encapsulated in hydrogel improved by incorporation of SAM (0.5 mg/ml). Furthermore, the migration of fibroblasts was facilitated in the scratched assay by SAM (0.5 mg/ml)/MSCs (1×10^5 ^cell/ml) conditioned medium. The GelMA hydrogel groupes revealed regeneration of full-thickness skin defects in rats after 3 weeks due to the high angiogenesis (6.3 ± 0.3), cell migration, and epithelialization.

**Conclusion:**

The results indicated in situ forming and tunable GelMA hydrogels containing SAM and MSCs could be used as efficient substrates for full-thickness wound regeneration.

## Introduction

Wound healing is a complex and vital process that requires effective treatment and timely coverage to ensure optimal functional outcomes, especially in full-thickness and extensive wounds (1–3). Skin tissue has self-regenerated properties, and endogenous repair begins immediately after damage (4). Platelet accumulation initiates the coagulation phase, which is followed by the migration of immune cells into the wound area, where they work with growth factors to remove germs and foreign bodies (5, 6). In the proliferation phase, fibroblasts and keratinocytes become engaged, resulting in tissue formation and epithelialization. Several intricate activities are occurring during this dynamic time, including the development of extracellular matrix (ECM), the production of soluble mediators, parenchymal cell proliferation, and migration (6, 7). However, increased immune cells, cell apoptosis, and growth factor distraction may cause the normal healing process to be interrupted or delayed in individuals with full-thickness wounds (8, 9). With the development of tissue engineering products, Advanced Therapeutic Medicinal products (ATMPs) have demonstrated success in skin tissue regeneration (10, 11). Additionally, cell-based and biological products enhanced the transfer of exogenous cells and molecules to the wound site and produced a proper and moist environment for the proliferation and migration of the native cells (10, 11). The viability of the encapsulated cells in the carrier, the type of carrier (12), mechanical and biological properties, and the choice of biomolecules and cytokines (13) for delivery to the wound site are still the main challenges and drawbacks of skin substitutes, and various studies have been conducted on this subject matter. In the early stages of tissue repair, cell-to-cell communication was regulated by growth factors (8). Proteomics analysis of amniotic membrane (AM) revealed a large range of growth factors, cytokines, proteases, soluble receptors, and other proteins (14, 15). The application of amniotic membrane was limited in the undermined, cavity, and deformed wounds (16, 17). A soluble form of AM has been shown to repair ocular damage and wounds today (16, 18). Indeed, according to AME proteomics analysis, a number of therapeutic growth factors have been approved for use in tissue regeneration (18–20). As tissue regeneration is a complex process and numerous elements were involved (19), we prepared a cocktail of soluble proteins derived from amniotic membranes. Exogenous signals are released by various growth factor panels in response to changes in their local environment (15). The solubilized amnion membrane’s total protein content may be quantified, and the optimum concentration in addition to the combination of SAM and cells in the fabrication of biological dressing can be assessed and optimized. Various hydrogel biomaterials have been recently investigated for applications in regenerative medicine, including as delivery systems for medications (21), proteins (22), and living cells (23). Natural hydrogels with minimal antigenicity, excellent biocompatibility, biodegradability, and affordability, like gelatin (i.e., hydrolyzed collagen), have gained increased popularity (24). Gelatin’s mechanical properties were improved when it was modified with methacrylate functional groups conjugated onto the amine or hydroxyl groups (25, 26). Affinity of hydrogel was further improved by the sulfated group (27, 28). Keratose, the oxidized form of keratin, may be appropriate for affinity-based drug delivery systems due to the presence of sulfonate functional groups (29). Most membrane and film dressings on the market are difficult to apply, so in situ formed dressings are preferable to self-prefabricated (30). In situ forming hydrogels that could contour wound defects have an advantage over the use of prefabricated hydrogel scaffolds as they would allow the dressing to conform to the wound without folding or nicking (31). A combination of tissue engineering and cell-based therapies is a promising approach for full-thickness wound healing (32). MSCs can produce biological factors modulating the immune response, proliferation, migration of keratinocyte and endothelial cells, and invocation of macrophages and endothelial progenitor cells to the wound area and cause angiogenesis (33). This study aims to develop and optimize an in situ forming, soluble amniotic membrane (SAM)-loaded cell-based hydrogel to promote full-thickness wound regeneration in rats.

## Materials and Methods

***Materials***

Lyophilized powdered gelatin from porcine skin (Type A, 9000-70-8), Dulbecco’s minimal essential medium (DMEM), Ham’s F12 medium (DMEM/F12,1:1), methacrylate anhydride (MA,760-93-0), penicillin/streptomycin, amphotericin B, fetal bovine serum (FBS), dimethyl sulfoxide (DMSO), hydrochloric acid (HCl), collagenase type II (17101015), 2-hydroxy-4′-(2-hydroxyethoxy)-2-methylpropiophenone (Irgacure 2959, Sigma 106797-53-9), 3-(4,5-dimethylthiazol-2-yl)-2,5-diphenyltetrazolium bromide (MTT reagent) and Trypan blue were purchased from Sigma-Aldrich (Dayaexir Co, Iran). The dehydrated amniotic membrane was supplied by Pishtaz Tebbe Abadis Co (Iran). keratose (KOS) was provided by Zharf Andishan Fanavar Zist Bespar Co (Iran). MTT and MTS Assay Kit (Cell Proliferation, Colorimetric) (ab197010) and Mouse/Rat Anti-CD31 antibody (ab28364) were purchased from Abcam. 

***Preparation of in situ forming cell-based SAM-loaded hydrogel***

*Synthesis of gelatin methacrylamide (GelMA)*

Porcine skin gelatin powder (Type A, 10% w/v) was dissolved in Dulbecco’s phosphate-buffered saline (DPBS) with stirring at 60 °C. Then, methacrylic anhydride (MA, 8.0 ml) was added to react with the gelatin solution with vigorous stirring at 50 °C for 3 hr. The reaction was stopped by diluting the polymer solution five-fold with hot DPBS (40 °C). The salts and unreacted MA were removed from the mixture by dialysis with a cut-off of 12-14 kDa in distilled water at 40 °C for 72 hr. White porous foam was then obtained by lyophilizing the solution for 48 hr and stored at -80 °C (34). 

*Preparation of soluble amniotic membrane*

The dehydrated amniotic membrane (DAM) was cut into small pieces, and soft powder was obtained by grinding frozen DAM with a miller machine. DAM powder (10% w/v) was dispersed in PBS (100 ml) and the pepsin enzyme (1% w/v) and HCl (0.01 mol/l) were added to the resultant solution, and then incubated for 48 hr at 4 °C. The DAM solution was neutralized with Sodium Hydroxide (5 mol/l) and centrifuged (18000 rpm) for 5 min. Subsequently, the supernatant was removed and proteins were stable by protease inhibitor (PI; 0.1% w/v). The solution was called solubilized AM (SAM) and was sterilized by filtering (0.2 µm) and kept at -80 °C (15).

*Fabrication of in situ forming cell-based SAM-loaded hydrogel*

**Figure 1:**
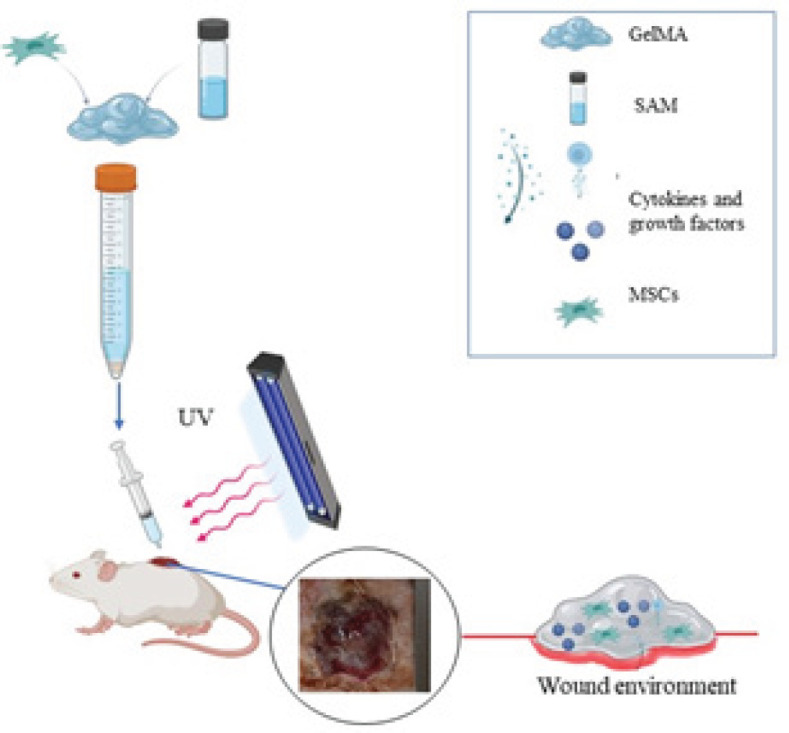
Fabrication SAM-loaded cell-laden hydrogel steps

GelMA lyophilized powder (10% w/v) was sterilized by gamma-ray irradiation (25 Gy). Subsequently, the sterile powder was dissolved in DPBS containing 0.5% w/v 2-hydroxy-1-4- (hydroxyethyl) phenyl) -2-methyl-1-propanone (Irgacure 2959); as a photoinitiator at 80 °C to reach final GelMA concentrations at 10% w/v. Furthermore, the SAM solution was diluted with DPBS (0.1, 0.5, 1, and 1.5 mg/ml), and the concentration was then optimized. For this aim, the ucMSCs (Pasteur Institute of Iran) were cultured to fourth passage, followed by short trypsinization, isolated cells (1× 10^5^ cells/ml) responded with SAM solution (0.5 mg/ml) and were added to GelMA prepolymer solution (Figure 1). The resultant solution was poured into the wound site with a 27-gauge syringe, instantly exposed to UV light (360–480 nm) for 60 sec. 

***Characterization of physical properties of GelMA hydrogels***

*FTIR analysis and determination of the crosslinked Component of GelMA*

GelMA and NpGel hydrogel were dried and materials were ground and subject to Fourier transform infrared spectrometry assessed at a resolution range of 400–4000 cm^-1^. Samples (10 mm diameter, 3 mm thickness) were fabricated upon UV (360-480) exposures for compression, swelling ratio, and degradation assessment. Non-photo-crosslinking Gelatin (NpGel) hydrogel was obtained as a control sample. GelMA and NpGel hydrogel was lyophilized for 48 hr and then the dry weight of the hydrogels was recorded (w0). Hydrogels were immersed in HCL) 10 mM, pH: 2) for 24 hr at 4 °C. Rehydrated hydrogel scaffolds were lyophilized again and the dry weights were recorded (w). This manner (35) was repeated three times, non-reacted soluble gel and residual weight was calculated as follows:

Remaining weight (%) =[(w0-w)/w0×100)

*Compressive mechanical analysis*

Photo-crosslinked hydrogel samples fabricated with or without cells or SAM, un-crosslinked were evaluated in terms of compressive mechanical properties with tested using a uniaxial compression, a universal electromechanical testing machine (Santam, STM-20, 60 N load cell) at a crosshead speed of 1 mm.min−1. The compressive modulus was calculated as the slope of the stress–strain curve at 10% strain.

*Biodegradation rate*

The degradation profile of hydrogels was assessed by incubation of hydrogel samples (d0) in the enzymatic environment. To this aim, hydrogels were soaked in Eppendorf tubes (1.5 ml) containing DPBS with type II collagenase (2 U ml-1) for 3 weeks, which adjusted to the collagenase concentration during wound healing (36). In order to maintain constant enzyme activity, the collagenase solution was refreshed every three days. At predetermined intervals, samples were washed with sterilized deionized water twice, freeze-dried, and their dry weight measured.

Degradation rate (%) =[(d0-d)/d0×100)

*Swelling ratio analysis*

Hydrogels were soaked in DPBS (pH: 7.4) at 37 °C for 24 hr. The filter paper was used to wipe off the excess water, and swollen weights of samples were recorded (s). The swelling ratio of the swollen gel was calculated according to the equation:

Swelling ratio (%) =[(S0-S)/S0×100)

*In vitro protein release assay*

SAM (0.5 mg/ml) was mixed with the hydrogels (GelMA 10% w/v, KOS 1% w/v). The SAM-loaded hydrogels were immersed in a buffer solution containing PBS for 7 days. 1, 3, 7, and 14 days were chosen as the test time intervals. The supernatants were removed and new buffer added at each interval. According to the manufacturer’s instructions, the manufacturer’s ELISA kit (R&D Systems) was used to measure the release of epidermal growth factor (EGF) from the SAM-loaded in the hydrogel. The following equation was used to determine the percentage of release:

Release (%) = amount of released EGF/ amount of total EGF in SAM

***Evaluation of cell viability in SAM-loaded hydrogel***

Different concentrations of SAM (0.1, 0.5, 1, and 1.5 mg/ml) were loaded within MSCs-laden hydrogel. Cell viability, proliferation, and efficacy of condition medium released from cell-laden hydrogel were assessed.

*MTS assay*

According to the manufacturer’s instructions, the MTS assay was used to measure the metabolic activity of the MSCs loaded within the hydrogels. Cells bioreduce the MTS chemical into a colorful formazan product that is soluble in the media used for cell culture. It is assumed that the NADPH or NADH produced by the dehydrogenase enzymes in metabolically active cells will be used for this conversion. The number of live cells in the culture directly relates to the amount of formazan product. The cells/hydrogel were rinsed with PBS after specified incubation intervals of 1, 3, and 7 days. The media was then changed to MTS solution (1:5 dilution in culture medium), which was then incubated in the dark for 3 hr at 37 °C and 5% CO2. The optical density (OD) was measured at 490 nm in an ELISA plate reader. 

*Quantification of DNA content*

In order to assess cell proliferation within the hydrogels, the total DNA content was evaluated. On day 14 of the culture, the cell-laden hydrogels had been totally homogenized and dissolved. The samples were processed with proteinase K (10 mg/ml) and lysis buffer (50 mmol/L tris-HCl, 50 mmol/L EDTA, 1 wt.% SDS, 10 mmol/L NaCl, pH 8.0) overnight at 65 °C. Then, using phenol/chloroform extraction and EtOH precipitation, DNA was removed from the aqueous phase. The resulting pellet was dissolved in water free of RNase, and we used a spectrophotometer to measure the amount of DNA at 280 nm. In terms of dry weight, the DNA concentration was given as g/mg of hydrogel.

Percentage of living cells = Average uptake of treated samples / Average uptake of control samples×100

*Effect of CM on fibroblast migration*

The conditioned medium prepared was assessed for in vitro wound healing. Briefly, human dermal fibroblasts (25 × 104 cells/well) were cultured on 6-well plates, after they achieved 80% confluency, they were scratched with a pipette tip along a straight line. After washing the cell debris with PBS, HDFs were treated with CM and a CM-free serum-free culture media as a negative control. A completed culture media (DMEM/FBS 10%) was supplied to the positive control. Digital pictures of the cells were acquired using an Olympus device at 0, 6, 12, and 24 hr after scratching. Image-J software was then used to evaluate the pictures. A vertical line was drawn to mark each well below the surface of the screen. The percentage of scratch closure was calculated as follows = [(S0-S)/S0) × 100, where S0 is the scratch area at time 0 and at is the scratch area at 6, 12, and 24 hr.

***In vivo wound healing study***

The Ethics Committee at AJA University of Medical Sciences approved all of the animal procedures in this research (6.5.201897000577), all animal procedures were performed in accordance with the guidelines for the care and use of laboratory animals. 8-week-old, Wistar rats (male) were used in this study. Each rat was anesthetized with an intraperitoneal injection of ketamine (80 mg/kg body weight) and xylazine (5 mg/kg). The hairs of the back were shaved and full-thickness wounds (2×2 cm) were created by skin excision. The defects were treated by AME-laden hydrogels (GelMA/AMEs), and cell-laden hydrogels (GelMA/AMEs/ucMSCs) for 21 days. The wounds in the control group were untreated. Sterile Vaseline gauze was used as a secondary dressing. The secondary dressing was changed every 3 days, the wound was cleaned with saline (0.9 wt.%). All animals received tramadol hydrochloride (3 mg/kg) and gentamicin (15 mg/kg) for 3 days postoperative. The health condition was monitored by a veterinarian. Animals were allowed to move freely in their cages and were fed a standard laboratory diet. The progress of healing was recorded by photograph on days 0, 7, 14, and 21. The rats were sacrificed after 3 weeks. For histological evaluation of healed skin, dissected samples were washed in PBS to remove blood and further fixed in formalin (10 wt.%) for two days and then immersed in paraformaldehyde (4 wt.%) at 4 °C. Subsequently, the tissues were embedded in paraffin, slide was stained with hematoxylin and eosin (H&E) and Masson Trichrome (MT). Finally, they were examined under a light microscope (Olympus BX51).

***Histological studies***

To stain the tissue sections for Immunohistochemistry, the sections were first deparaffinized in xylene, rehydrated via EtOH gradients, and then treated with an antigen retrieval solution containing sodium citrate buffer (pH 6), at a temperature of 90 °C, for 30 min. The sections were then blocked with 10% goat serum (v/v). Primary antibodies for CD31 were stained with Abcam (ab34712) (1:200 dilution), followed by secondary antibodies for IgG (Invitrogen (A11001, A11008, 1:500 dilution).

***Statistical analysis***

All experiments were repeated at least 3 times and results were expressed as Mean ± SD. Kruskal Wallis Rank Sum Test was used (an alternative to the non-parametric version ANOVA test) to compare the median of outcomes across groups. After a statistically significant Kruskal Wallis test, Dunn’s test was used for several pairwise comparisons, and adjusted P-values were used to account for multiple tests (Bonferroni approach) and P<0.05 was considered statistically significant across all statistical tests. All analyses were performed with R Statistical Software (R Core Team 2022 Version 4.2.2).

## Results

***Characterization of Gelatin methacrylate (GelMA) and prepared hydrogels***

*Fourier transform infrared spectrometer analysis*

**Figure 2:**
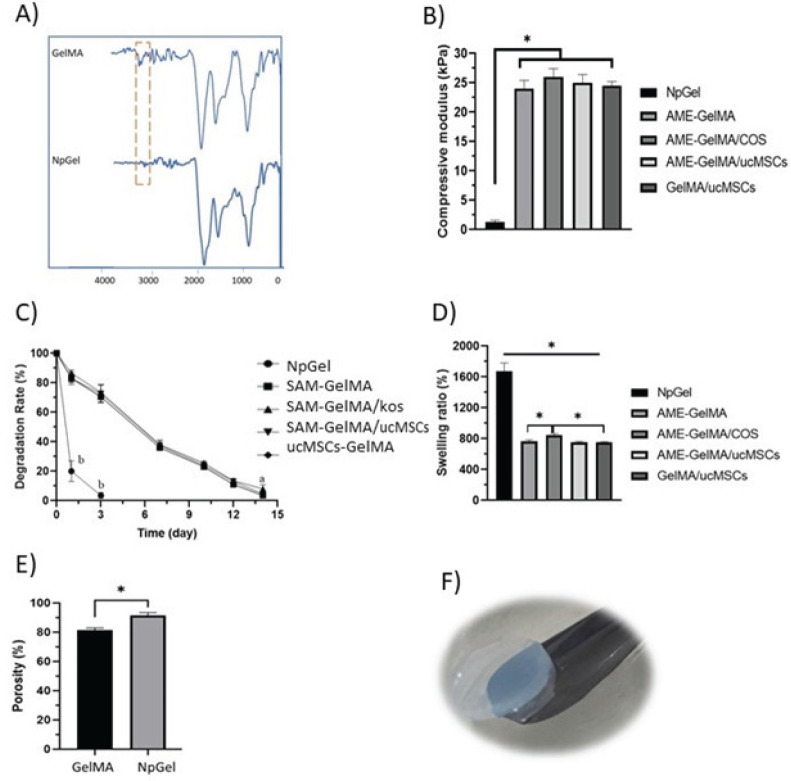
Synthesis and Physical characterization of gelatin methacrylate hydrogels (GelMA)

As shown in FTIR data, the GelMA (SAM-loaded) showed clear peak changes from NpGel approximately 3000 cm^-1^ (Figure 2A). The amide B band was observed at 3078 cm^-1^ (37), indicating that MA and gelatin were successfully cross-linked through the formation of an amide bond. The FTIR data demonstrated that, although physically combined, GelMA and SAM may also create amide bonds by photo-crosslinking. This unique structure cannot be broken apart without disintegrating covalent connections, distinguishing it from interpenetrating networks that comprise two or more distinct networks (38). It was demonstrated by the mechanical characteristics test that GelMA created a sturdy structure.

*Analysis of the mechanical properties of hydrogels*

The compressive modulus rate in NpGel and GelMA hydrogels was 2 kPa and 26 kPa, respectively (Figure 2B). Since the NpGel hydrogel has only physical crosslinks, it showed a low compressive modulus, which was expected. However, GelMA hydrogel showed a higher compressive modulus, which prevents its contraction in the culture medium.

*Degradation profile of hydrogels*

The stability of hydrogels was evaluated by degradation assay during 7 days in the enzyme solution. Collagenase enzyme is a protease that consists of a polypeptide chain and is capable of digesting collagen fibers (36). Gelatin covalent bonds in GelMA hydrogel can reduce the rate of biodegradation in the presence of collagenase. Due to the absence of chemical bonds, NpGel hydrogel showed faster degradation in comparison with GelMA hydrogel. The weight loss of NpGel hydrogel (81.8±2.1%) on the first day in comparison with the GelMA sample (13.7±1.9%) indicated the greater stability of chemically crosslinked gelatin against enzymatic degradation. Figure 2C shows that the gelatin hydrogel is completely degraded within 3 days in the enzyme solution, while the remaining weight of GelMA was calculated (63.7±1.3%).

*Swelling behavior and porosity of hydrogel*

The swelling rate of NpGel physical hydrogel (868.3±25.1%) indicated a significant increase in comparison with GelMA chemical hydrogel (730.8±20.3%) (Figure 2D). Indeed, GelMA swelling reduction was attributed to the tough structure resulting in chemical bonds between the methacryloyl gelatin chains. Furthermore, methacryloyl bonds, which have little hydrophilicity, replace the hydrophilic amine functional group in the polymer chain (39). Furthermore, gelatin hydrogel water absorption increased porosity and open network structure of hydrogel. In contrast, increasing the density of gelatin crosslinking resulted in the formation of smaller pores throughout the GelMA hydrogel. Since porosity is necessary for cell migration and nutrient exchange, the porosity of hydrogels was measured by the liquid displacement method. The porosity rate was calculated in NpGel (93.5±1.3%) and GelMA hydrogel (24.8±1.5%) (Figure 2E). These results showed that the porosity range of hydrogels is suitable for maintaining cell migration, proliferation, and nutrient exchange.

*Determination of crosslinked component of GelMA*

**Figure 3:**
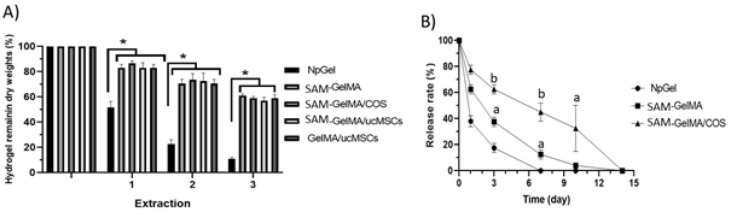
Release behavior of epidermal growth factors

The relative degree of crosslinking of the hydrogel after methacrylate was assessed by Acid-soluble gel. It should be minimal when all the free groups of gelatins are crosslinked together except for extractable gelatins. As shown in Figure 3A, all the remaining GelMA mass was 68.2±2.21% after a 3-time soak in HCL solution, while the non-crosslinked sample was 10.25±0.14%. These results showed that photo-crosslink of modified gelatin was successful and most gelatin chains participated in this reaction, further supporting FTIR information.

*Analysis of the SAM protein from hydrogel in vitro*

The *in vitro* release of EGF from SAM-GelMA, SAM-GelMA/COS, and NpGel groups was assessed for 14 days. As shown in Figure 3B, there was significantly less cumulative release of EGF from the SAM-GelMA/KOS (22±0.7%) hydrogel in comparison with the SAM-GelMA hydrogel (41.5±0.4 %) in day 1. Also, there was significantly less cumulative release of EGF from the SAM-GelMA group compared with the NpGel.

*Optimization of the SAM dosage*

**Figure 4:**
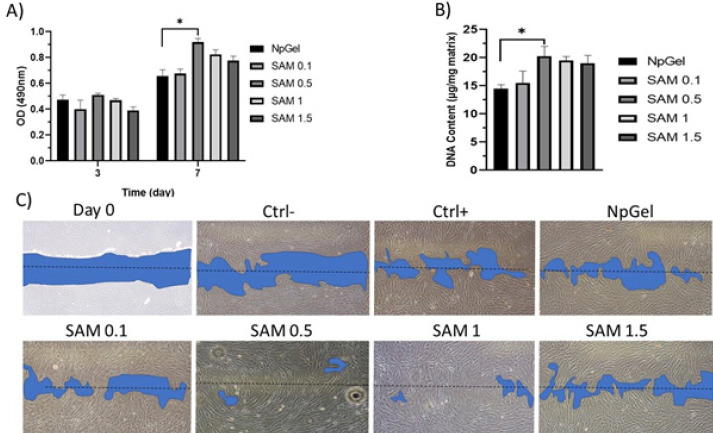
Cytocompatibility of cell-laden hydrogels

Metabolic activity of MSCs-laden SAM-loaded hydrogels was assessed by MTS assay on days 1 and 7 (Figure 4a). All samples revealed good cytocompatibility during 7 days; each group had significant increases in OD from Day 3 to Day 7. Interestingly, the OD in MSCs-laden hydrogels loaded with SAM (0.5 mg/ml) significantly increased on day 7 compared with the control group (0.92±0.08 vs 0.65±0.04). Quantification of DNA content within 7 days from cell seeding has been used to assess *in vitro* proliferation. As shown in Figure 4B, the DNA content of MSCs-laden hydrogels loaded with SAM (0.5 mg/ml) group significantly increased compared with the other hydrogel (21.2±1.5 vs 13.8±1.4 µg/ml matrix). Conditioned medium prepared from cell-laden SAM-loaded hydrogel was assessed in in vitro migration assay during 12 hr (Figure 4C). According to the microscopic images of the scratch area, cell migration was observed after 3 hr in all groups. The scratch area in the wells treated with SAM (0.5 mg/ml) group (95±3.1%) was decreased compared with positive Ctrl (65±2.7%) and NpGel (67.1±2.4%) groups after 12 hr.

*Wound morphology and hydrogel scaffold fate*

**Figure 5:**
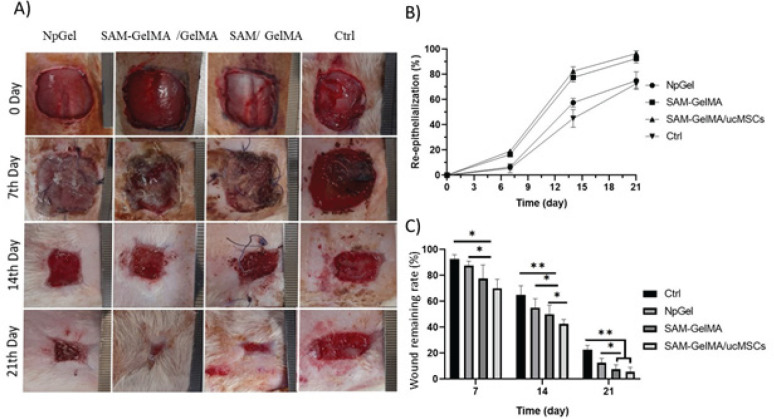
Macroscopic images of rat wound healing

GelMA hydrogels were observed for 10 days at the wound site. Then they were replaced with new dermal and epithelial tissue. Gelatin hydrogel was seen on the wound during 6 days, and in the control group scab tissue was observed at the control (without treatment) wound site (Figure 5A).

*Re-epithelialization*

The rate of epithelial tissue formation of the healed skin is defined as a percentage in each time period. As shown in Figure 5B, there was no significant difference between different groups during the 3 days, but the formation of epithelial tissue in the SAM-GelMA/ucMSCs group on days 7, 14, and 21 showed a significant increase compared to other groups. The epithelial tissue on day 7 was much higher in the SAM-GelMA group (44.7 ± 0.79%) compared to the control group (26.6 ± 0.12%). The epithelialization in the SAM-GelMA/ucMSCs group (77.8±1.13%) and SAM-GelMA (69.3±0.97%) showed a significant increase compared to the control group (51.6 ± 1.18%) on day 14.

*Wound healing rate*

Wound area reduction in the SAM-GelMA and SAM-GelMA/ucMSCs groups was significantly higher compared with the positive control group (NpGel) and negative control (Figure 5C), the progress of wound healing was obvious after 7 days. Wound remaining in the SAM-GelMA/ucMSCs group (3.2±0.21%) was less than other control groups (25.1±1.31%), SAM-GelMA (8.2 ± 1.25%). 

***Histological studies***

**Figure 6:**
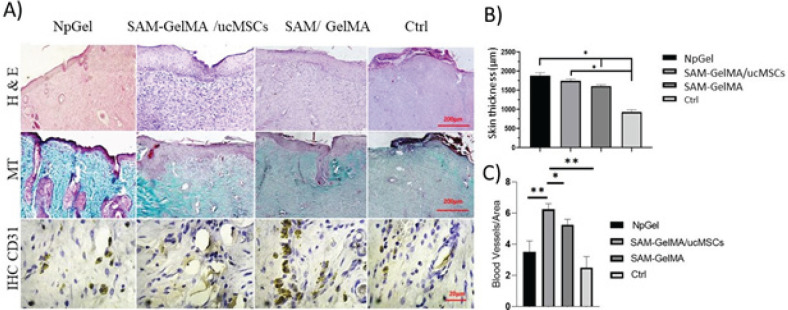
Morphological evaluation of rat skin lesions with histological analysis method

Wound healing is a temporary process that requires the coordinated activity of many factors such as growth factors and cytokines, extracellular matrix, blood vessels, and different types of cells (8). In order to evaluate the infrastructure of skin and wound healed with SAM-GelMA and SAM-GelMA/ucMSCs groups, H&E staining was performed. The surface area of the wound was evaluated by collecting a series of individual images using ImageJ software in one image (Figure 6A). This evaluation shows that the thickness of the new skin in the control group is thinner than in the treatment groups (Figure 6B). The appearance of hair in the repaired skin and tissue appendages such as hair follicles and sweat glands was observed in the central area of the wound, which was attributed to the migration and proliferation of endogenous cells. Infiltration of macrophages was observed in all groups on day 3. While, in SAM-GelMA and SAM-GelMA/MSCs due to the strong anti-inflammatory activity of SAM, less inflammation was observed than in the control group. In addition, angiogenesis was observed in the granulation tissue and in SAM-GelMA and SAM-GelMA/MSCs, which can be attributed to the presence of SAM and angiogenesis-stimulating growth factors such as VEGF. As reported, the presence of blood vessels is necessary for granulation tissue formation, because it carries out oxygenation, nutrition, and removal of waste materials. Fibroblast infiltration and neovascularization increased on days 7 and 14, and wound healing appeared. In the study of MT staining, it was observed that the amount of fibrosis in wounds treated with SAM-GelMA and SAM-GelMA/MSCs groups is lower compared to the control group. In addition, the collagen fibers in the SAM-GelMA/MSCs group were organized in a more distinct arrangement, while these fibers were denser and thicker in parallel in the control group. 

***Angiogenesis***

The density of blood vessels was determined by counting the number of blood vessels in different parts of the tissue using high-resolution microscopic images (Figure 6C). The number of blood vessels in the SAM-GelMA/MSCs group (6.3±0.3) was significantly increased compared with the control group (2.2 ± 0.2, Figure 6B).

MSCs and SAM loaded in GelMA hydrogel. Prepolymer hydrogel was poured in situ on the wound created on the back of the rat

SAM: Solubilized amniotic membrane

A) Fourier transform infrared spectrometer analysis of GelMA. B) Compressive modulus of hydrogels; *<0.05. C) Degradation behavior of the hydrogels during 14 days in DMEM/F12 medium containing (100 μg/ml collagenase enzyme) a: *P*<0.05, b: *P*<0.01. C) GelMA hydrogel. D) Swelling profile during 24 hr; *<0.05. E) Porosity of hydrogels (*<0.05(. F) GelMA hydrogel. NpGel; Non-photo-crosslinking Gelatin, SAM-GelMA; Soluble amniotic membrane loaded in gelatin methacrylates, SAM-GelMA/KOS; Soluble amniotic membrane loaded in gelatin methacrylate conjugate by keratose, SAM-GelMA/uuMSCs; Soluble amniotic membrane loaded in gelatin methacrylate contain umbilical cord mesenchymal stem cell

A) Determination of chemical modification of photo-crosslinkable GelMA; *<0.05. (B) Cumulative EGF release from hydrogels over 14 days (a, b). Significant difference between SAM-GelMA/KOS and the other groups; a: *P*<0.05, b: *P*<0.01); kOS, Kerateose

(A) MTS assay of the Mesenchymal stem cells/Solubilized AM (MSC/SAM) encapsulated in the hydrogels at different time points. A significant difference between the optical density [OD] of SAM 0.5 compared with NpGel-MSCs (without SAM); *<0.05. (B) Quantification of DNA content within 7 days from cell culture; *<0.05. (C) Microscopic images from HDFs morphology treated with different CM and control groups; Ctrl -: conditioned medium (CM) free DMEM, Ctrl +: FBS/DMEM, SAM 0.1:CM prepared from SAM 0.1 mg/ml, and MSCs (1×10^5^ cell/ml) loaded in the hydrogel; HDFs: human dermal fibroblast

A) Digital images were provided after the 3rd, 7th, 14th, and 21st day of injury. B). Wound remaining (٪) was calculated by dividing the remaining wound surface by the surface of the original wound size. The GelMA group accelerated wound closure C): Re-epithelialization of the wound was calculated by measuring the residual wound area and contraction by measuring the new epithelialization skin of the wound; * <0.05, ** < 0.01.

A) H&E and MT staining shows the epidermis and dermis structure. B) Healed skin thickness measures revealed that treated skin, both with SAM-GelMA/ucMSCs and SAM-GelMA, was thicker than untreated wounds. C) Quantification of blood vessel density by counting the number of vessels with high-resolution microscopic images; *<0.05, **<0.01; MT, Masson's trichrome stain; H&E, Hematoxylin and eosin stain.

## Discussion

Currently, stem cells and biological sources such as cell secretory (40,41) or tissue-derived structural substances (42) are used to regenerate damaged tissue, further providing an alternative to conventional methods or a successful complementary therapy for wound healing (42). In this regard, researchers developed innovative products that are based on cells (43, 44), cell-derived products such as conditioned media secreted from cell culture loaded in three-dimensional scaffolds (42) and therapeutic properties were evaluated for the fabrication of new skin substitutes. Although previous study indicates that the amnion membrane contains an array of growth factors, proteins, and cytokines (45) that have been shown to promote skin regeneration and wound healing, there is still a major limitation to the delivery and application of this intact amnion to extensive or irregularly shaped wounds (46, 47). This refers to the majority of treatment conditions where an amnion sheet does not adjust an atypical wound size or topology, or where fast degradation because of poor mechanical properties has resulted in the development of a new generation of amnion-derived products (28, 48, 49). The present cell-laden SAM-loaded pre-polymer solution was injected into the wound site, and following exposure to light, it quickly crosslinked to conform toward the wound contour (34). Additionally, its mechanical, degrading, and biological properties may be readily tuned by adjusting the degree of methacrylamide modification, GelMA concentration, or photopolymerization duration (28, 34). Mechanical properties results illustrated that the compression module and degradation rate of cell-laden SAM-loaded GelMA hydrogel significantly increased after photo-crosslinking in comparison with non-photo-crosslinked gelatin hydrogel (Figure 2B). As shown by Zhao et al., higher mechanical properties and slower degradation rates could be obtained at the modification of methacrylamide, GelMA (10 %) 26±3 kPa and 30±2 %, day 3, respectively (34). Our study met the GelMA (10 %) mechanical properties (34). Higher water absorption in NpGel hydrogel led to enhanced porosity and a more open network topology (50). On the other hand, smaller porosity and higher mechanical properties developed due to increased crosslinking density (39). Fabrication of wound dressing based on gelatin by freeze-drying method revealed higher porosity and mechanical properties (51). The unique characteristics of gelatin are maintained when it is modified with photocrosslinkable methacrylamide groups (GelMA), but the hydrogel additionally exhibits more mechanical properties in comparison with freeze-drying method (51, 52). Crosslinked GelMA kept about 63% of its initial mass whereas the non-cross-linked sample (Figure 5). The crosslinked network was determined using FTIR by integrating peaks that correspond to the aromatic residues of gelatin and double bonds that correspond to methacrylamide. Growth factors loading in the scaffold may be advantageous for regenerating cell-based tissues and could be useful for MSCs support epithelialization (53). It has been demonstrated that sulfated glycosaminoglycan can stabilize keratose-bound growth factors through electrostatic interactions between negatively charged sulfate groups as well as positively charged amino acid residues of growth factors (29,54). The growth factors may be sustained for a long time as a result of this physical binding. SAM has been entrapped in GelMA hydrogels; its release via UV–vis spectroscopy was determined at 278 nm (Figure 3B). The cumulative release of EGF from GelMA hydrogel functionalized by KOS (1 Wt.%) was significantly lower in comparison to that of GelMA and gelatin hydrogels. Since the chemical structures of the SAM and GelMA hydrogel both include an aromatic segment, the EGF could be bound non-covalently to the GelMA-KOS. As a result, GelMA/KOS hydrogel may sustain SAM release with variable molecule sizes more effectively than KOS-free GelMA (Figure 3B). According to Momeny et al., 34.5% of the total SAM protein release occurred within the first 48 hr (19), but Murphy et al. observed that SAM protein bulk release occurred within the first week (20). SAM-GelMA/KOS additionally reduced the cumulative release of the SAM proteins. Our previous study (45) indicated that Amniotic membrane Extract (AME) prepared by sonication method, concentration of 1 mg/ml, significantly increased fibroblast proliferation and migration which aligned with the results reported by Momeny *et al.* (19). While, in the current study, the concentration of 0.5 mg/ml increases the proliferation and migration of fibroblast and MSCs loaded in the hydrogel. The OD of NpGel loaded with only MSCs revealed a considerable increase on day 3. On day 7, it showed a significant decrease when compared to the group that had cell-laden GelMA/SAM 0.5 mg/ml. The result can be attributed to the hydrogel scaffold contracting and cell death caused by the accumulation of cells (42). Human dermal fibroblast (HDF) migration is greatly increased by condition medium generated by MSCs-laden SAM-loaded hydrogel when compared to another group. This is supported by the paracrine activity of SAM and MSCs, which is consistent with the research conducted (55) to assess the effect of AME on cell ucMSCs culture (55). According to a study’s results, ucMSCs conditioned medium was migrating HDF (56). We demonstrated that both the SAM-loaded GelMA and SAM-loaded GelMA hydrogels with encapsulated MSCs could facilitate the repair of rat skin defects, which was presumably due to prolonged release and better retention of SAM and cytokine released from ucMSCs. The morphological analysis of the cell-laden SAM-loaded GelMA hydrogel group greatly increased the percentage of wound closure by re-epithelialization and reducing wound contraction. In the histology evaluations, the GelMA group contained cells and SAM revealed thicker healed skin and more neovascularization generally (57). Fibroblast growth factor (FGF-family), epidermal growth factor receptor (EGF-R), epidermal growth factor (EGF), and vascular endothelial growth factor (VEGF), all of which are involved in the formation of new blood vessels, were significantly increased in the cell-laden SAM-loaded GelMA group compared to other groups (58, 59). Indeed, this study supports the effects of GelMA on survival, fibroblast activity, and prevention of wound contraction as well as the effect of SAM and MSCs together to boost growth factors that are useful in wound healing. The cell-laden SAM-loaded GelMA hydrogel supports the proliferation and migration of fibroblasts in in vitro and in vivo conditions. The rat full-thickness wound model and its repair are commonly used in basic research to evaluate potential therapies for wound repair. However, there are major limitations to these models that are well known, such as not being similar to humans, endogenous repair of rats, and wound repair without any therapeutic measures. However, on days 3, 7, 14, and 21 in this study, we compared the effect of epithelialization, contraction, and percentage of wound healing in the groups. Considering that we found that there are significant changes in these parameters, we predict that this improvement will be much more effective in chronic wounds and wounds that have a slow healing process. Also, it is difficult to evaluate the long-term scar due to the rapid closure of the wound in the healthy rats used in this study. 

## Conclusion

 This study was designed in order to fabricate and evaluate the in situ SAM-loaded cell-laden hydrogel with the controlled drug release ability for the regeneration of full-thickness skin defects in rats. According to the results, the mechanical properties of the photo-crosslinkable hydrogel, degradation in the presence of collagenase enzyme (13.7 ± 1.9%), and compression module (26 kPa), were increased. In this regard, conjugation of a methacrylate functional group onto the hydroxyl or amine groups of gelatin was confirmed by Fourier transform infrared (FTIR) spectroscopy at a resolution of 3078 cm^-1^. The viability and proliferation of ucMSCs loaded in the photo-crosslinkable hydrogel were increased in the presence of the SAM (0.5 mg/ml) during 7 days after culture. We demonstrated that incorporation of keratos (1%) with high affinities to biological agents could stabilize and improve the sustained release of SAM (22±0.7%). CM prepared from hydrogels containing SAM and MSCs can increase the migration of fibroblasts in in vitro conditions. In this regard, SAM (0.5 mg/ml) revealed the greatest effect on migration of fibroblasts compared to other concentrations of SAM (0.1,1 and 1.5 mg/ml). GelMA scaffold prevented wound contraction, and the simultaneous presence of SAM and ucMSCs increases the rate of healing, angiogenesis, and wound closure. This in situ hydrogel can be used as a new skin substitute for rapid wound repair and regeneration and further studies in the clinical environment.

## Authors’ Contributions

M AA and Z J conceived, designed, and conducted the experiment. M AA wrote the manuscript. A E re-analyzed the statistical data and results. MA H re-analyzed the data and edited the manuscript.

## Funding

Department of Tissue Engineering and Trauma, AJA University of medical science, Tehran, Iran provided Grant-in-Aid for Scientific Research.

## Conflicts of Interest

No declarations of interest are reported by the authors.
